# Neutrophil Paralysis in *Plasmodium vivax* Malaria

**DOI:** 10.1371/journal.pntd.0001710

**Published:** 2012-06-26

**Authors:** Fabiana Maria de Souza Leoratti, Silvia Cellone Trevelin, Fernando Queiroz Cunha, Bruno Coelho Rocha, Pedro Augusto Carvalho Costa, Humberto Doriguêtto Gravina, Mauro Shugiro Tada, Dhelio Batista Pereira, Douglas Taylor Golenbock, Lis Ribeiro do Valle Antonelli, Ricardo T. Gazzinelli

**Affiliations:** 1 Laboratório de Imunopatologia, Centro de Pesquisas René Rachou, Fundação Oswaldo Cruz, Belo Horizonte, Minas Gerais, Brazil; 2 Departamento de Farmacologia, Faculdade de Medicina de Ribeirão Preto, Universidade de São Paulo, Ribeirão Preto, São Paulo, Brazil; 3 Departamento de Bioquímica e Imunologia, Universidade Federal de Minas Gerais, Belo Horizonte, Minas Gerais, Brazil; 4 Centro de Pesquisas em Medicina Tropical de Rondônia, Porto Velho, Rondônia, Brazil; 5 Division of Infectious Diseases and Immunology, University of Massachusetts Medical School, Worcester, Massachusetts, United States of America; Federal University of São Paulo, Brazil

## Abstract

**Background:**

The activation of innate immune responses by *Plasmodium vivax* results in activation of effector cells and an excessive production of pro-inflammatory cytokines that may culminate in deleterious effects. Here, we examined the activation and function of neutrophils during acute episodes of malaria.

**Materials and Methods:**

Blood samples were collected from *P. vivax*-infected patients at admission (day 0) and 30–45 days after treatment with chloroquine and primaquine. Expression of activation markers and cytokine levels produced by highly purified monocytes and neutrophils were measured by the Cytometric Bead Assay. Phagocytic activity, superoxide production, chemotaxis and the presence of G protein-coupled receptor (GRK2) were also evaluated in neutrophils from malaria patients.

**Principal Findings:**

Both monocytes and neutrophils from *P. vivax*-infected patients were highly activated. While monocytes were found to be the main source of cytokines in response to TLR ligands, neutrophils showed enhanced phagocytic activity and superoxide production. Interestingly, neutrophils from the malaria patients expressed high levels of GRK2, low levels of CXCR2, and displayed impaired chemotaxis towards IL-8 (CXCL8).

**Conclusion:**

Activated neutrophils from malaria patients are a poor source of pro-inflammatory cytokines and display reduced chemotactic activity, suggesting a possible mechanism for an enhanced susceptibility to secondary bacterial infection during malaria.

## Author Summary

*Plasmodium vivax* is responsible for approximately 60–80% of the malaria cases in the world, and contributes to significant social and economic instability in the developing countries of Latin America and Asia. The pathogenesis of *P. vivax* malaria is a consequence of host derived inflammatory mediators. Hence, a better understanding of the mechanisms involved in induction of systemic inflammation during *P. vivax* malaria is critical for the clinical management and prevention of severe disease. The innate immune receptors recognize *Plasmodium sp*. and initiate a broad spectrum of host defense mechanisms that mediate resistance to infection. However, the innate immune response is the classic “two-edged sword”, and clinical malaria is associated with high levels of circulating pro-inflammatory cytokines. Our findings show that both monocytes and neutrophils are highly activated during malaria. Monocytes produced high levels of IL-1**β**, IL-6 and TNF-**α** during acute malaria. On the other hand, neutrophils were a poor source of cytokines, but displayed an enhanced phagocytic activity and superoxide production. Unexpectedly, we noticed an impaired chemotaxis of neutrophils towards an IL-8 (CXCL8) gradient. We proposed that neutrophil paralysis is in part responsible for the enhanced susceptibility to bacterial infection observed in malaria patients.

## Introduction

Malaria is a complex disease that affects approximately 300 million people every year. Among the different *Plasmodium* species that infect humans, *P. falciparum* is the main cause of deaths in sub- Saharan Africa. On the other hand, *P. vivax* is responsible for approximately 60–80% of the malaria cases in the world [1], [2], and contributes to significant political, social and economic instability in the developing countries of Latin America and Asia [1].

The innate immune system recognizes *Plasmodium* sp. by different pattern-recognition receptors and initiates a broad spectrum of defense mechanisms that mediate host resistance to infection [3], [4]. However, the innate immune response is the classic “two-edged sword”, and clinical malaria is associated with high levels of circulating pro-inflammatory cytokines. The outcome of infection depends on a balance between pro- and anti-inflammatory responses allowing the formation of an effective immune response, while limiting its pathogenic potential [5], [6], [7].

*Toll like receptors* (TLR) play an important role in recognition of pathogens through distinct pathogen-associated molecular patterns (PAMPs). Activation of TLR on monocytes, dendritic cells and neutrophils can induce changes in the expression of surface proteins and release inflammatory mediators such as cytokines and chemokines. The production of cytokines amplifies innate immune responses and shapes the development of acquired immunity. In addition, activated myeloid cells release high levels of reactive oxygen species (ROS) and antimicrobial peptides that efficiently kill invading pathogens [8], [9].

It is noteworthy that glycosylphosphatidylinositol anchors and DNA from *Plasmodium* parasites are important PAMPs that activate TLR during malaria [10], [11], [12], [13]. Some *in vitro* studies show that phagocytosis of opsonized hemozoin (HZ) decreases expression of HLA-DR in monocytes [14], [15]. On the other hand, study has demonstrated that DNA bound to HZ induces monocytes to produce high levels of cytokines and contribute to dendritic cell maturation [16]. While other report evaluated activation of polymorphonuclear cells and observed elevated levels of myeloperoxidase, lysozyme and lipocalin in patients with severe malaria [17], the involvement of neutrophils in malaria pathogenesis has been poorly investigated.

Our findings show that both monocytes and neutrophils are highly activated during malaria. Monocytes produced high levels of IL-1β, IL-6 and TNF-α in response to TLR agonists during acute malaria and seem to be the main source of pro-inflammatory cytokines in the blood. On the other hand, neutrophils were a poor source of cytokines, but displayed an enhanced phagocytic activity and superoxide production. Interestingly, we noticed an enhanced expression of G-protein receptor protein kinase (GRK2) associated with decreased levels of CXCR2 and impaired chemotaxis of neutrophils towards an IL-8 (CXCL8) gradient. Our findings indicate a mechanism by which malaria patients may become more susceptible to bacterial infection.

## Methods

### Ethics statement

All protocols and consent forms were approved by the Institutional Research Board from University of Massachusetts Medical School (IRB-UMMS 10268), the Ethical Committees on Human Experimentation from Centro de Pesquisa em Medicina Tropical (CEP-CEPEM 095/2009) and Centro de Pesquisas René Rachou – Fundação Oswaldo Cruz (CEP-CPqRR 2004), as well as by the National Ethical Committee (CONEP 15652) from Ministry of Health, Brazil. A signed informed consent was obtained from each subject prior to enrollment in the study.

### Patients

Patients were recruited and examined at CEPEM in Porto Velho, a malaria endemic area in the Amazon region of Brazil. Up to 100 ml of peripheral blood was collected immediately after confirmation of *P. vivax* infection by thick blood smear film and 30–45 days after chemotherapy (n = 26, ranging from 18 to 66 years old [35±9.5]). Patients were treated for 10 days with chloroquine and primaquine. *P. vivax* infection was confirmed by polymerase chain reaction (PCR) analysis [18]. The clinical manifestations of malaria were fever, myalgia, chills, arthralgia, nausea, vomiting or diarrhea, but no patient had complicated malaria. Peripheral blood was also collected from 15 healthy donors (HD) ranging from 21 to 56 years old [32±8] living Porto Velho and negative for *P. vivax* infection by thick blood film and PCR. All the experiments were done using fresh cells.

### Cellular immunophenotyping

Whole blood was stained with antibodies from ebioscience: anti-CD14-FITC (clone 61D3), anti-TLR2-PE (clone T2.5), anti-TLR4-PE (clone HTA125), anti-HLA-DR-PE (clone L243), anti-CD14-APC (clone 61D3), anti-CD62L-APC (clone DREG56); BD Bioscience-Pharmingen: anti-CD66b-FITC (clone G10F5), anti-CD88-PE (clone C85-4124), CXCR2-PE (clone 6C6), anti-CD16-PercpCy5.5 (clone3G8), anti-CD15-FITC (clone HI98); R&D: CCR2-PE (clone 48607) Subsequently, red blood cells (RBC) were lysed with FACS lysing solution (BD Biosciences) following manufacturer's instructions, washed with phosphate buffered saline (PBS) and maintained in paraformaldehyde 2% (PFA) until the acquisition on an FACScan upgraded with a second laser (5 colors). The software used for acquisition was CellQuest Pro from BD and Rainbow from Cytek. Data analyzed using FlowJo version 9.3.2 (TreeStar)

### Purification of monocytes and neutrophils

Peripheral blood mononuclear cells (PBMC) and neutrophils were enriched on-site by gradient centrifugation over Ficoll-Paque™ plus (GE-Healthcare). Isolated PBMC were washed twice in RPMI, ressuspended at 5×10^7^/ml in PBS supplemented with 2% heat- inactivated fetal bovine serum (FBS) and 1 mM EDTA. Monocytes were purified from PBMC with the Human Monocyte Enrichment Kit (STEMCELL-Technologies) following manufacturer's instructions. The cell layer containing the granulocytes were collected just above the red blood cells phase and the cell suspension was lysed with NH_4_Cl 0.15 M with KHCO_3_ 0.1 M and Na_2_EDTA 0.1 mM. Cells were ressuspended in PBS (2% FBS, 1 mM EDTA) at final concentration 5×10^7^/ml. Purification of neutrophils were performed with the Human Neutrophil Enrichment Kit (STEMCELL-Technologies) following manufacturer's instructions. Purity of monocytes and neutrophils was measured by flow cytometry after staining with mAbs specific for CD14, CD66b and CD16. Purified monocytes were CD14^+^CD16^−^CD66b^−^ and neutrophils were CD14^−^CD16^+^CD66b^+^. Cell preparations reached over 98% of purity and viability of 90–100%.

### Tissue culture assays

Purified monocytes and neutrophils were plated in 96-well cell culture plates at a final concentration 2×10^5^/well in RPMI1640 (Sigma Aldrich R6504) (10% FBS and 100 µg/ml streptomycin/100 U/ml penicillin) in the presence or absence of *Toll like receptors* agonists: 100 ng/ml lipopolysaccharide (LPS - TLR4 agonist, Sigma-Aldrich) or 100 ng/ml Pam2CSK4 (Pam - TLR2/TLR1 agonist, InvivoGen). Supernatants were harvested after 48 hours of culture and kept at −20°C until cytokine measurement.

### Cytokine measurements

IL-8 (CXCL8), IL-1β, IL-6, IL-10 and TNF-α were quantified in cryopreserved plasma and supernatants from monocyte and neutrophil cultures using the Cytometric Bead Array kit (CBA, BD Biosciences Pharmingen) following manufacturer's instructions. The limits for cytokine detection were 3.6 pg/ml (IL-8), 7.2 pg/ml (IL-1β, 2.5 pg/ml (IL-6), 3.3 pg/ml (IL-10) and 3.7 pg/ml (TNF-α).

### Phagocytosis and superoxide assays

Phagocytosis was assessed by incubating neutrophils for 20 min with opsonized zymosan (5×10^6^ particles/cell) in a glass slide. Cells were stained by the May-Grünwald-Giemsa (Rosenfeld) method (Laborclin Pinhais, PR, BR). The number of cells containing phagocytic vacuole was determined by counting 100 neutrophils. Nitro Blue Tetrazolium (NBT) assay was performed using peripheral blood. Briefly, 25 µl of blood sample were added to the same volume of NBT (1 mg/ml) at 37°C for 15 min and an additional 15 min at room temperature. Percentage of cells reducing NBT was established by counting 100 neutrophils.

### Chemotaxis assay

Chemotaxis was performed with purified neutrophils using a 48-well microchamber (NeuroProbe, Gaithersburg, MD) with a 5 µm pore polycarbonate membrane. Fifty thousand neutrophils were allowed to migrate toward IL-8 (CXCL8) (10 ng/ml), CCL2 (1 ng/ml), or medium alone. After 1 h incubation, at 37°C with 5% CO_2_ the membrane was removed, fixed and stained. The number of neutrophils that migrated through the membrane was counted under a light microscope on at least 5 randomly selected fields.

### Real-Time PCR

Total RNA from neutrophils was extracted using the Trizol reagent (Invitrogen, Carlsbad, CA). Total RNA to cDNA was carried out by reverse transcription (Superscript II, Gibco Life Technologies Grand Island, NY). Real Time PCR (qPCR) analysis of CXCR2 receptor mRNA profile was performed with the Platinum SYBR® Green qPCR SuperMix-UDG kit (Invitrogen-Life Technologies, USA) and the reactions were processed in ABI Prism 7500 equipment (Applied Biosystems, Warrington, UK) was performed in an ABI Prism 7500. The reactions were carried out in a final volume of 10 µl containing 6.25 µl of SYBR Green, 0.5 µM of each primer (designed on the basis of the mRNA sequence of the receptor gene available at the GenBank) and 1 µl of cDNA. RT-PCR cycles were 95°C for 15 s and 60°C for 1 minute and the dissociation curve was constructed by increasing temperatures from 60°C to 90°C. The primers used were: CXCR2 sense: 5′- AGATGCTGAGACATATGAATTT -3′ and CXCR2 antisense: 5′- CTTTTCTACTAGATGCCGC-3′, β-Actin sense: 5′-GCTCGTCGTCGACAACGGCTC -3′ and β-Actin antisense: 5′- CAAACATGATCTGGGTCATC -3′. The expression of β-actin mRNA was used as a control for all samples. The relative level of gene expression was determined by the comparative threshold cycle (Ct) method using the formula −2^ΔΔCt^ whereby data for each sample were normalized to β-actin mRNA levels.

### Immunofluorescence assay for GRK2

Neutrophils purified on glass slides were fixed with 4% paraformaldehyde in a wet chamber at room temperature. Cells were fixed with 4% paraformaldehyde, permeabilized with 0.2% Triton X-100 (Amersham Pharmacia Biotech, CA) and blocked with 1% bovine serum albumin (Sigma-Aldrich) in PBS containing normal goat serum (1∶50). Cells were then incubated overnight with polyclonal rabbit anti-GRK-2 antibody (1∶200, clone C-15, Santa Cruz Biotechnology) followed by incubation with red fluorescent Alexa Fluor 594 (goat anti-rabbit IgG (H+L); 1∶400, Life Technologies). Nuclear material was stained with 4, 6-diamidino-2-phenylindole (DAPI; Sigma-Aldrich). Images of stained cells were captured using an epi-fluorescence microscope (BX-40; Olympus, Japan). The mean fluorescence intensity was determined from a linear measurement of fluorescence of individual cells using Image J software (Imaging process and analysis in Java, National Institute of Mental Health). Ten cells of five randomly chosen fields of each slide were analyzed.

### Statistical analysis

Statistical analysis was performed using Prism software, version 5.0 (GraphPad). The results were analyzed using two-tailed paired test. Wilcoxon testing was used when data did not fit a Gaussian distribution. The results were analyzed using unpaired t test when two groups were compared. Mann-Whitney (MW) test was used when a normality test failed. Differences were considered to statistically significant, when *p*≤0.05.

## Results

### High levels of circulatory cytokines in *P. vivax* malaria patients

**Figure 1:**
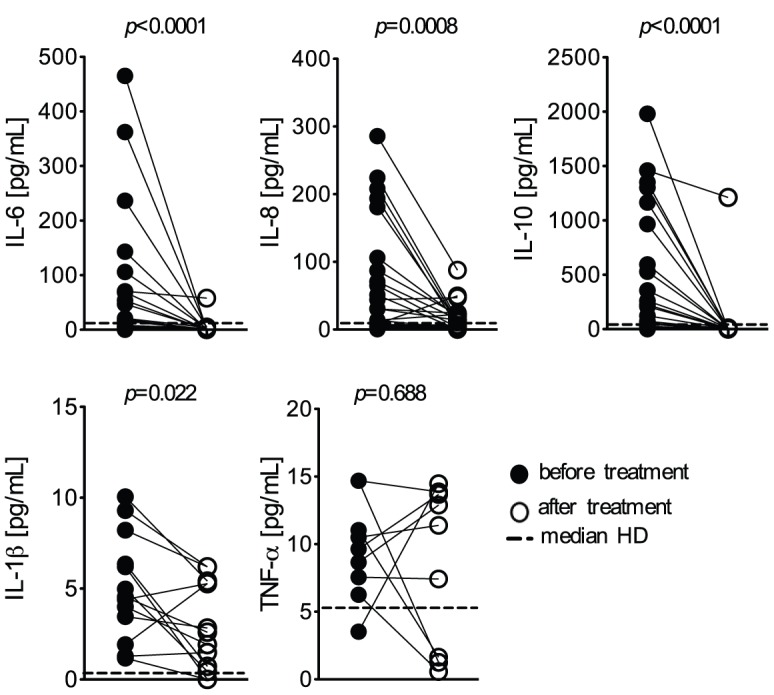
High levels IL-1β IL-6, IL-8 and IL-10 in plasma from patients infected with *P. vivax*. The cytokines IL-8 (CXCL8), IL-1β, IL-6, IL-10, TNF-α were measured in the plasma of *P. vivax*-infected subjects (n = 26), before (closed circles) and 30–45 days after treatment (open circles). Dotted lines represent medians of given measurements from healthy donors (HD; n = 13). Levels of cytokines were measured employing the Cytometric Bead Array (CBA). Significant differences are indicated with *p*-values using Wilcoxon signed rank test when the data failed the normality test.

High levels of the pro-inflammatory cytokines, IL-1β, IL-6, IL-8, were found when comparing the sera from the same patients presenting the active disease and after anti-malarial therapy and parasitological cure or to HD. (**Figure 1**). The levels of TNF-α in the sera did not differ significantly. The levels of IL-10 were also higher before treatment than after parasitological cure. No significant differences in the level of circulating cytokines were found between HD (IL-8 median: 9.8; interquartile (IQR): 1.8- 21; IL-1β median: 0.2 IQR: 0.13–2.8; IL-6 median: 0.14; IQR:0–5; IL-10 median: 0–7.1; IQR: 0–7 and TNF-α median: 5.4; IQR 0–21) and malaria patients who had completed the chemotherapy.

### Monocytes and neutrophils activation during *P. vivax* infection

**Figure 2:**
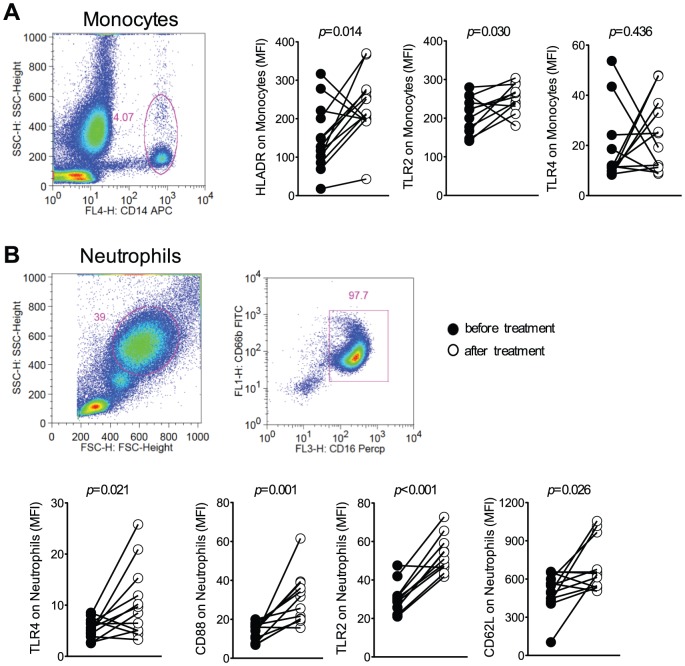
Systemic activation of monocytes and neutrophils during *P. vivax* malaria. Mean fluorescence intensity (MFI) of HLA-DR, TLR2 and TLR4 was evaluated on monocytes (A) and CD62L, CD88, TLR2 and TLR4 on neutrophils (B) in whole blood from *P. vivax-*infected subjects (n = 12), before (closed circles) and 30–45 days after treatment (open circles). Representative density plots showing the gate strategy for monocytes CD14+ (A) and neutrophils CD66b+CD16+ (B) are shown. Significant differences are indicated with *p*-values using Wilcoxon signed rank test when the data failed the normality test.

The expression of various activation markers was analyzed on monocytes (**Figure 2A**) and neutrophils (**Figure 2B**). Monocytes were gated taking in consideration the expression of CD14, as shown in the representative density plot (**Figure 2A**). Lower expression of HLA-DR (median fluorescence intensity-MFI) was observed on monocytes from 9 out of 12 *P. vivax*-infected patients, when compared to the same patients after anti-malarial treatment. To assess the neutrophil population, CD66b^+^CD16^+^ cells were gated. As CD66b is GPI-anchores highly glycoslated protein, widely expressed on human neutrophils and to regulate neutrophil acitvation. Lower levels of CD62L (8 out of 12 subjects) and CD88 (all patients) were found on neutrophils from malaria patients before treatment (**Figure 2B**). The lower expression of HLA-DR as well as CD62L is a cell adhesion molecule, and CD88 is a G protein-coupled receptor for C5a, have been previously described in activated monocytes and neutrophils, respectively [14], [19]. The levels of these activation markers return to normal levels (comparable to the median of HD (monocytes: HLA-DR (median: 278; IQR: 143–323); neutrophils: CD88 (median: 27; IQR: 17–32) and CD62L (median: 601; IQR: 439–666); in cells from *P. vivax*-patients submitted to anti-malarial therapy.

### Monocytes are a major source of cytokines during *P. vivax* malaria

**Figure 3:**
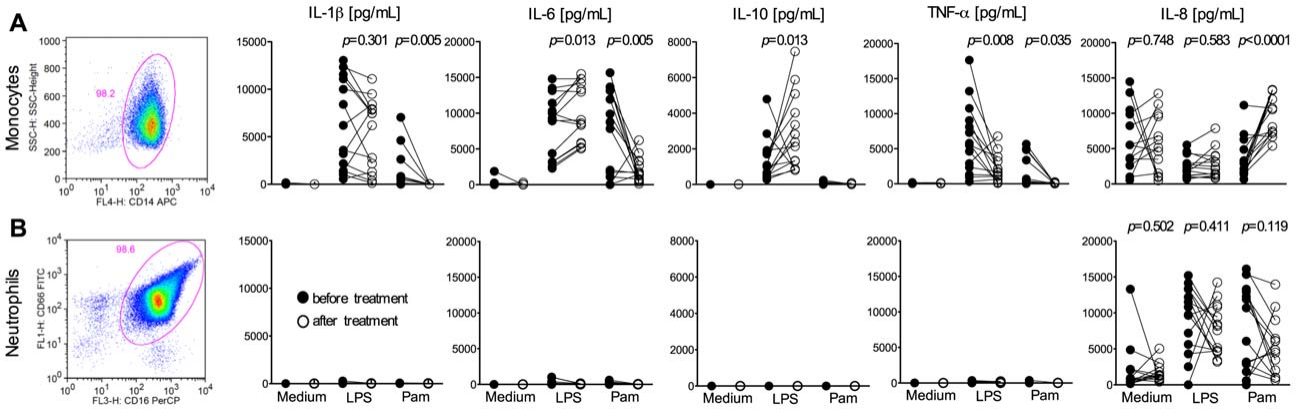
TLR agonists induce production of high IL-1β, IL-6 and TNF-α levels by monocytes from *P. vivax*-infected subjects. Purified monocytes (A) or neutrophils (B) from *P. vivax-*infected subjects before (closed circles; n = 13) and 30–45 days after treatment (open circles; n = 13) were cultured for 48 hours in the absence or presence of LPS or Pam. Levels of IL-1β, IL-6, IL-10, TNF-α, and IL-8 (CXCL8) were measured in supernatant of monocyte (A) and neutrophil (B) cultures. Levels of cytokines were measured employing the Cytometric Bead Array (CBA). Significant differences are indicated *p*-values using paired t test or Wilcoxon signed rank test when a normality test failed.

The expression of TLR2 was lower on both monocytes (8 out of 11 subjects) and neutrophils (10 out of 11 subjects) from malaria patients, whereas expression of TLR4 was only affected on neutrophils (8 out of 12) from infected individuals (**Figures 2A and 2B**). After parasitological cure, the surface expression of TLR returned to levels comparable to cells from HD (monocytes: TLR2 (median: 256; IQR: 241–276) and TLR4 (median: 11; IQR: 9–17; neutrophils: TLR2 (median 46; IQR: 36–62) and TLR4 (median: 3.5; IQR: 2.9–6.4)). Two millions per ml (2×10^5^/well) of highly purified monocytes or neutrophils were cultured with or without TLR agonists and cytokines measured in the tissue culture supernatants (**Figure 3A and B**). Cell preparations reached over 98% of purity, as shown in the representative dot plots presented in **Figure 3**. Except for IL-8, neutrophils were over all a poor source of cytokines. No detectable levels of IL-1β, IL-6, IL-10 and TNF-α were found in culture supernatants of neutrophils stimulated or not with LPS or Pam (**Figure 3B**), even when neutrophils were used at a concentration of 1×10^7^/ml (1×10^6^/well). On the other hand, upon stimulation with LPS or Pam, high levels of IL-1β, IL-6, and TNF-α were produced by monocytes from malaria patients. (**Figure 3A**). Interestingly, the levels of LPS-induced IL-10 were lower in culture supernatants of monocytes derived from patients with ongoing *P. vivax* infection. After parasitological cure the cytokine production by monocytes returned to levels comparable to those produced by monocytes from HD (median: 1692; IQR: 1200–5265). IL-8 (CXCL8) was the only cytokine produced in high levels by neutrophils. Despite the down-modulation of TLR2 and TLR4, stimulation with TLR agonists increased the levels of IL-8 produced by neutrophils (**Figure 3B**) from malaria patients before (medium versus LPS, *p* = 0.0010 or Pam, *p* = 0.0024) and after treatment (medium vs LPS, *p* = 0.0002; or Pam, *p* = 0.0017). IL-8 was also produced by monocytes and the levels of cytokine did not differ between patients, before and after treatment (**Figure 3A**).

### Enhanced oxidative and phagocytic activity by neutrophils from malaria patients

**Figure 4:**
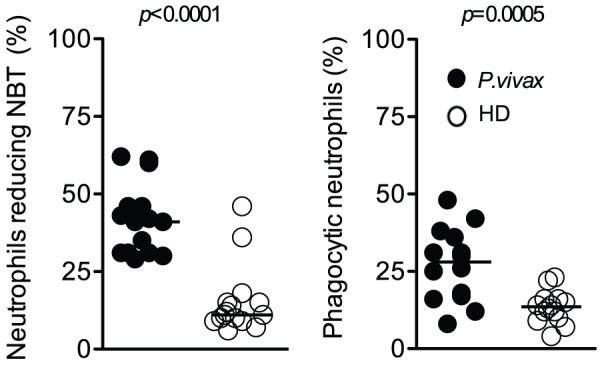
Neutrophils from *P. vivax*-infected patients produce high levels of superoxide and display enhanced phagocytic function. Neutrophils were isolated from *P. vivax*-infected patients (closed circles; n = 15) or healthy donors (open circles; n = 15), and the frequencies of neutrophils reducing NBT (left panel) as well as cell containing zymosan (right panel) were quantified. Significant differences are indicated with *p*-values using unpaired t test or Mann-Whitney test when a normality test failed.

The O_2_^−^ production by neutrophils was increased during acute malaria (**Figure 4**). Higher percentage of NBT positive cells was observed in the blood of *P. vivax*-infected patients (**Figure 4** **, left panel**). The function of neutrophils was further investigated by assessing their phagocytic activity, which was also higher in cells from *P. vivax*-infected individuals (**Figure 4** **, right panel**).

### Impaired chemotaxis by neutrophils from malaria patients

**Figure 5:**
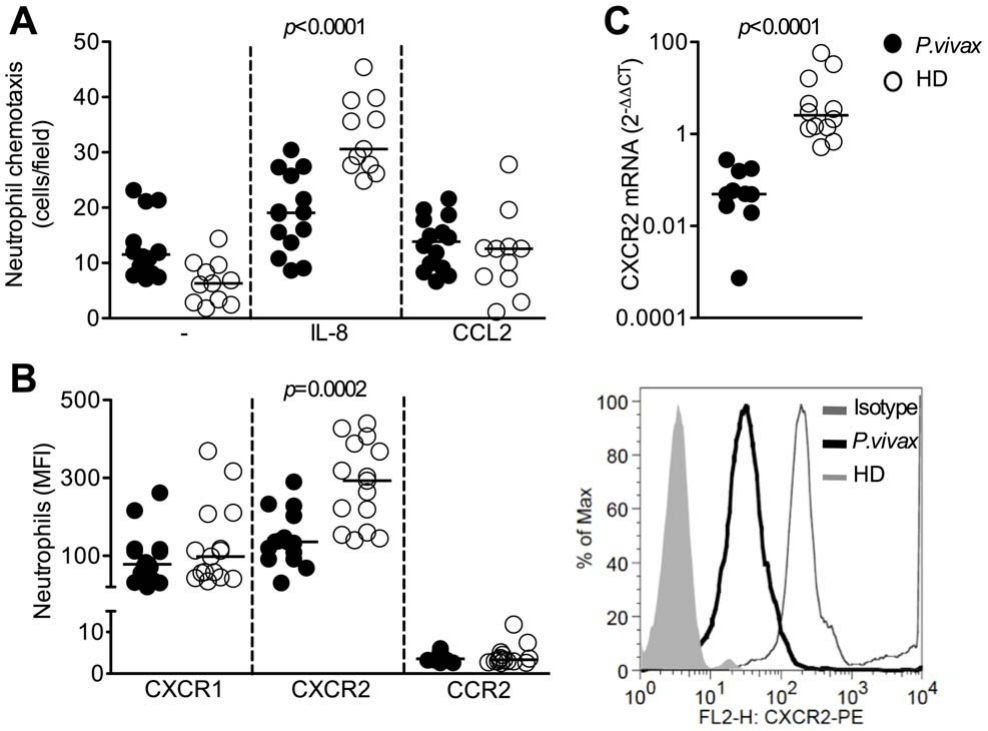
Malaria impairs neutrophils response to CXCR1 and CXCR2 ligand. Neutrophils were isolated from *P. vivax*-infected patients (closed circles; n = 15) or healthy donors (open circles; n = 15), and chemotaxis towards IL-8 (CXCL8) and CCL2 was assessed (A). MFI of CXCR1, CXCR2 and CCR2 on neutrophils were evaluated by flow cytometry and representative histograms of CXCR2 expression are shown (B). CXCR2 message was measured by qPCR (C). Significant differences are indicated with *p*-values using unpaired t test or Mann-Whitney test when a normality test failed.

The ability of purified neutrophils to migrate towards CXCR1, CXCR2 and CCR2 ligands (IL-8 and CCL2) was evaluated by using a modified Boyden chamber assay [20]. Neutrophils from malaria patients showed impaired chemotaxis to IL-8, when compared to HD (median: 31; IQR: 28–39) (**Figure 5A**). The poor migration of neutrophils from malaria patients was associated with decreased expression of the chemokine receptor CXCR2, as indicated by qPCR (**Figure 5C**) and FACS (**Figure 5B**). Chemotaxis to CCL2 and expression of CCR2 by neutrophils were minimal and not altered in malaria patients (**Figures 5A and 5B**).

### Enhanced expression of GRK2 in neutrophils from malaria patients

**Figure 6:**
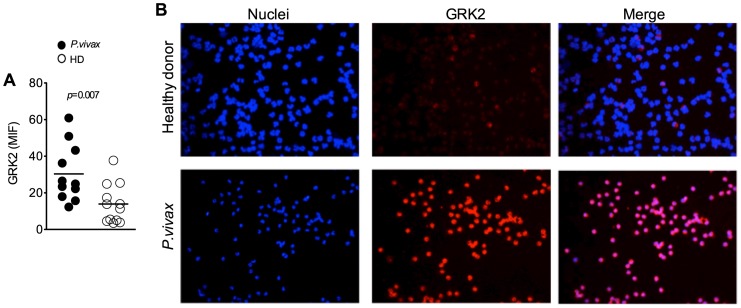
GRK2 expression is enhanced in neutrophils during acute malaria. Neutrophils isolated from *P. vivax*-infected patients (closed circles; n = 11) or healthy donors (closed circles; n = 12) were stained for GRK2 and mean fluorescence intensity (MFI) of GRK2 was quantified (A). Representative fluorescence microscopy illustrating GRK2 expression in neutrophils from a healthy donor and a *P. vivax*-infected patient (B). Significant difference is indicated with *p*-values using unpaired t test.

GRK2 has been described to down-modulate expression of chemokine receptors. Importantly, continuous and excessive activation of neutrophils results in an increased expression of GRKs, which phosphorylate G protein–coupled receptors (GPCR) leading to receptor desensitization [21]. GRK2 expression was measured by immunofluorescence in neutrophils purified from blood of *P. vivax*-infected patients and HD (median: 12.6; IQR: 4.7–23). Compared with HD, a significant increase in GRK2 expression was observed in neutrophils from *P. vivax*-infected patients (**Figure 6A and 6B**).

## Discussion

The pathogenesis of *P. vivax* malaria is a consequence of host derived inflammatory mediators [1], [22]. Hence, a better understanding of the mechanisms involved in induction of systemic inflammation during *P. vivax* malaria is critical for the clinical management and prevention of severe disease. Neutrophils are the most abundant leukocyte population in the peripheral blood and thus being in direct contact with red blood cells, the main cells population that is parasitized by *Plasmodium* species. Surprisingly, very few studies have addressed the role of the polymorphonuclear cells (PMNs) during malaria. In this study, we investigated the activation and function of neutrophils during acute episodes of *P. vivax* malaria. Neutrophils were shown to be highly activated presenting enhanced phagocytic activity as well as superoxide production. In contrast, neutrophils were found to be poor sources of cytokines during malaria. It is noteworthy that we described for the first time an impaired chemotaxis by circulatory neutrophils from malaria patients. Importantly, different studies have described an association of severe malaria and bacterial infections [23], [24]. Thus, our results suggest that the failure of neutrophils to migrate to the sites of infection may represent an important mechanism that leads to enhanced susceptibility of malaria patients to secondary bacterial infections.

We first noticed that both circulatory neutrophils (decreased expression of CD62L and CD88) and monocytes (decreased expression of HLA-DR) from malaria patients were highly activated. We then asked, what would be the primary source of pro-inflammatory cytokines during *P. vivax-*malaria? We found that in the blood, monocytes are an important source of cytokine compared with neutrophils. Despite the fact that neutrophils are an important source of cytokines [25], we found that except for IL-8 (CXCL8), the neutrophils from malaria patients produced none or very small amounts of pro-inflammatory cytokines (*i.e.*, IL-1β, IL-6, and TNF-αin response to TLR agonists. Importantly, PBMCs from individuals experimentally or naturally infected with *P. falciparum* are hyperresponsive and produce high amounts of pro-inflammatory cytokines once activated with TLR agonists [6], [7]. Here, we observed that highly purified monocytes (but not neutrophils) derived from *P. vivax* malaria patients were primed and produce high levels of IL-1β, IL-6 and TNF-α upon TLR stimulation. In contrast, the monocytes from malaria patients produced low levels of IL-10, even when activated with TLR agonists. Thus, we favor the hypothesis that during malaria monocytes differentiate into an inflammatory stage producing high levels of pro-inflammatory cytokines and low levels of IL-10.

On the other hand, circulatory neutrophils from malaria-patients displayed enhanced phagocytic activity and constitutively released high levels of superoxide. The process of neutrophil activation could involve phagocytosis of opsonized parasites, which would in turn trigger the antibody dependent respiratory burst [26]. Alternatively, phagocytosis of the malaria pigment (hemozoin) may also activate neutrophils [27]. The enhanced effector function of neutrophils may account for a more efficient up-take and destruction of free parasites and infected erythrocytes [26]. On the other hand, activated neutrophils have been shown to cause damage of endothelial cells, in a process that is mediated by sera of malaria patients [28]. Therefore, enhanced effector functions in neutrophils could be involved in both host resistance and pathogenesis of *P. vivax* malaria.

Unexpectedly, we found an altered migration towards IL-8 gradient, which was associated with a decreased expression of CXCR2 on neutrophils from *P. vivax*-infected patients. Functional studies showed that upon phagocytosis of bacteria by neutrophils there is reduction in expression of CXCR1 and CXCR2 [29]. Down-regulation of CXCR2 in severe sepsis also results in failure of neutrophil migration that is associated with enhanced susceptibility to bacterial infection [30]. Furthermore, expression of CD88 (C5a receptor) and CD62L (L-selectin) is decreased on the surface of neutrophils from *P. vivax*-malaria patients. CD88 is a G protein-coupled receptor involved in recruitment [19], [31], whereas CD62L is a key molecule that mediates cytoadherence of leukocytes [32]. Thus, altogether, our data strongly suggest that systemic activation neutrophils, leads to failure of extravasation and chemotaxis from blood to the tissues. Since IL-8 mediates chemotaxis and stimulate neutrophils to release specific granules and proteases to fight microbial infections [33], [34], the impairment of neutrophil migration to the site of infection would prevent front line cells to promote an inflammation to effectively kill infectious pathogens allowing secondary infections.

Production of IL-8 has been assessed in several cell type upon stimulation, several medical conditions and even constitutively [35], [36], [37]. The observed high levels of circulatory IL-8 may mediate desensitization and/or down-regulation of CXCR2 in acutely infected malaria patients. In addition, TNF-α [38], nitric oxide [30], heme-oxygenase products [39] and TLR ligands [20], cause the heterologous desensitization of CXCR2 via GRK2 induction. As previous described in *vivax* malaria [40], [41], we did not find a high level of TNFα in serum, the monocyte stimulated with TLR agonists produced high amounts of TNF-α that may contribute to CXCR2 desensitization. In addition, *Plasmodium* can be recognized by TLR2, TLR4 and TLR9 [10], [42] and induce down-regulation of CXCR2 via GRK2. Importantly, CD88 is also desensitized via GRK2 [43]. Thus, decreased expression of CXCR2 and CD88 on neutrophils from malaria patients may be a consequence of an enhanced expression of GRK2.

GRKs constitute a group of serine/threonine protein kinases that are key modulators of protein-coupled receptor signaling (GPCR) [44]. A major mechanism for desensitization of activated GPCR is their phosphorylation by GRKs [45]. Of note both CD88 and CXCR2 are GPCRs. Deficient expression of GRK and regulation of chemokine receptors in GRK2^+/−^ mice results in enhanced migration of lymphocytes and chemotaxis toward CCL4, the CCR5 ligand [46]. It was also described that transcription of GRK2 and GRK5 is upregulated upon LPS-mediated activation, leading to reduced expression of chemokine receptor and neutrophils chemotaxis [47]. GRK2 and GRK5 expression are enhanced in sepsis patients [48] and in rodent models of severe sepsis [20], which are associated with impaired migration of neutrophils and enhanced susceptibility to secondary microbial infection.

For many years, *P. vivax* malaria was considered a benign and self-limited disease, especially when compared to *P. falciparum* infection [49]. However, recent studies highlighted the association of *P. vivax* malaria with life-threatening manifestations, such as respiratory distress, severe thrombocytopenia and anemia, as well as neurological manifestations [1], [22], [50], [51], [52], [53], [54]. A main hypothesis of our research group is that secondary infections, in malaria primed individuals, is a main cause of severe disease. In this regard, pro-inflammatory priming during malaria would result in dramatic decrease in the threshold to initiate a septic shock [7], due to an over-reaction to secondary infections, particularly in the case of bacteria that have extremely potent TLR agonists.

Importantly, areas of the world with the highest incidence and prevalence of malaria also have a high incidence of bacterial infections, including *Salmonella*, *Pneumococcus* and *Meningococcus* [23], [55]. Furthermore, a recent study highlights that severe malaria as indicated by respiratory distress, anemia and mortality, is 8.5 times more elevated in children with both malaria and bacteremia as compared to infection with *P. falciparum* alone [24]. Thus, co-infection with bacteria is not only common, but as we might predict, it is an important factor influencing outcome of disease and development of severe disease [24], [56]. Here, we demonstrate for the first time that circulatory neutrophils from malaria patients display a decreased expression of chemokine receptors and adhesion molecules, which culminates in impaired chemotaxis. Hence, our results suggest that a failure of these PMNs to migrate to peripheral tissues is an important mechanism leading to enhance susceptibility to bacterial infection during malaria.
